# Opposing serial effects of stimulus and choice in speech perception scale with context variability

**DOI:** 10.1016/j.isci.2024.110611

**Published:** 2024-07-30

**Authors:** Carina Ufer, Helen Blank

**Affiliations:** 1 Department of Systems Neuroscience, University Medical Center Hamburg-Eppendorf, 20246 Hamburg, Germany; 2 Hamburg Brain School, University Medical Center Hamburg-Eppendorf, 20246 Hamburg, Germany

**Keywords:** Neuroscience, Behavioral neuroscience, Social sciences

## Abstract

In this study, we investigated serial effects on the perception of auditory vowel stimuli across three experimental setups with different degrees of context variability. Aligning with recent findings in visual perception, our results confirm the existence of two distinct processes in serial dependence: a repulsive sensory effect coupled with an attractive decisional effect. Importantly, our study extends these observations to the auditory domain, demonstrating parallel serial effects in audition. Furthermore, we uncover context variability effects, revealing a linear pattern for the repulsive perceptual effect and a quadratic pattern for the attractive decisional effect. These findings support the presence of adaptive sensory mechanisms underlying the repulsive effects, while higher-level mechanisms appear to govern the attractive decisional effect. The study provides valuable insights into the interplay of attractive and repulsive serial effects in auditory perception and contributes to our understanding of the underlying mechanisms.

## Highlights

• Disentangling stimulus and choice history reveals opposing effects on perception
• Choice history attracts and stimulus history repels current speech perception
• Repulsive stimulus history effect only revealed after accounting for choice history
• Stability in speaker context strengthens repulsive effects of stimulus history

## Graphical abstract

**Figure:**
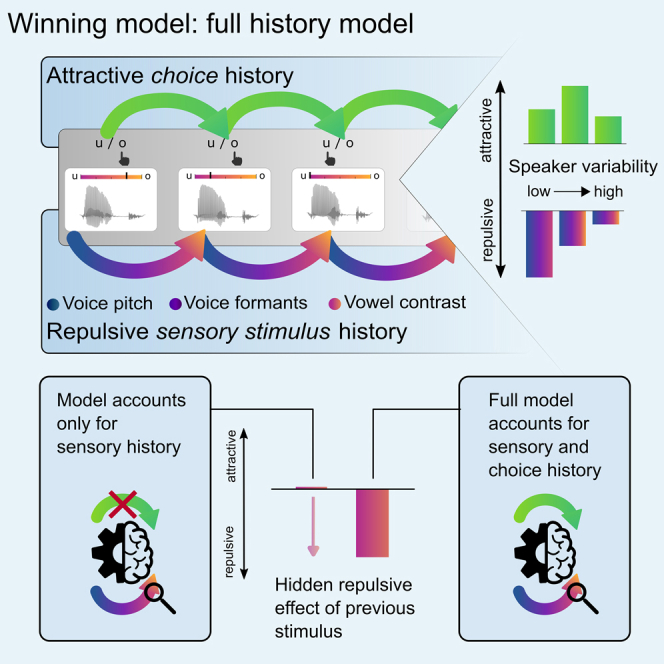

## Introduction

Perception of incoming sensory information is profoundly influenced by preceding sensory input. In the visual domain, numerous studies have provided evidence that visual perception tends to be biased by previously encountered similar stimuli, a phenomenon labeled as serial dependence.^1^^,^^2^^,^^3^^,^^4^ However, while serial effects should also be of great importance in audition, as the auditory signal inherently unfolds in the temporal dimension,^5^ reports of serial dependence in the auditory domain are sparse (but see^6^^,^^7^). Serial dependence has been reported in a positive direction, i.e., attracting the current percept toward the previous stimulus (attractive effect^8^^,^^9^^,^^10^), and in a negative direction, i.e., repelling the current percept away from the previous stimulus (repulsive effect^11^^,^^12^^,^^13^^,^^14^). These opposing serial effects have been explained with the properties of the presented stimuli. It has been suggested that repulsive effects are more likely to result from exposure to strong, salient, high-contrast, and long-duration stimuli, whereas brief, less salient, and low-contrast stimuli are associated with attractive effects.^1^ Attractive and repulsive stimulus history effects may serve different goals: while repulsive serial dependence, akin to adaptation-like processes, might help to weight previous information about the recent past to maximize sensitivity to change,^15^^,^^16^^,^^17^^,^^18^ attractive serial dependence might follow a dynamic form of integration whereby the tendency of the physical world to remain stable or change continuously is exploited.^3^^,^^10^

As attractive serial dependencies of the previous stimulus have been observed over many perceptual dimensions — for example, for olfactory stimuli^19^ along with various visual features like orientation,^10^ motion,^20^ numerosity,^6^^,^^21^ position,^22^ gender,^23^^,^^24^ age,^24^ face attractiveness,^25^ food palatableness,^26^ and art aesthetics^27^ — it has been argued that it constitutes a domain-general mechanism. This ubiquity led to the hypothesis that serial dependence serves as a functional mechanism integrating similar stimuli over time to maintain perceptual coherence as our external world exhibits temporal stability. However, the view that attractive serial dependence leverages to maintain a continuous perceptual experience has recently been challenged.^28^ If attractive serial dependence serves to smooth out variations in sensory inputs, the attractive effect should scale with the statistical variability of the environment. Thus, in contexts with high temporal correlations between stimuli, i.e., low variability, the current stimulus should be perceived as stable through attraction to the previous percept. However, contrary to this expectation, a recent study discovered that such correlations across visual stimuli repelled the current sensory input away from the previous one.^28^ Interestingly, in contrast to the repulsive serial dependence of the stimulus history, an attractive serial effect of the previous choice was observed throughout different temporal correlations.

Crucially, in experiments that observed attractive serial dependence from the previous stimulus, effects by the response were not always controlled for.^6^^,^^19^^,^^25^^,^^26^ Thus, an attractive dependence from the previous choice could superimpose an effect overshadowing the potential repulsive serial dependence from the stimulus history. Indeed, when the previous response was taken into consideration, many experiments identified this attractive serial effect of choice.^14^^,^^29^^,^^30^^,^^31^^,^^32^^,^^33^^,^^34^ Moreover, a recent re-analysis of published data revealed that the initially found attractive serial dependence from the previous stimulus could be the result of an attractive decisional process overshadowing a repulsive effect of the previous stimulus.^35^ With this mounting evidence that two opposing history effects of stimulus and choice counteract each other, we tested these effects in the lesser-studied auditory domain. Specifically, the current study aimed to investigate how previous stimulus features (here speech and voice) together with the previous choice influence the perception of the current auditory stimulus in different contexts of environmental variability.

While temporal context effects have a long-standing history in auditory perception,^36^^,^^37^^,^^38^^,^^39^ they have little impact on the research field of serial dependence. Our experiment bridges the gap between those two disciplines. Most studies investigating context effects on auditory perception have focused on negative effects, where the current percept is repelled away from the previous sound.^40^ These studies typically used inducer sounds that did not require any decision and were manipulated to elicit a repulsive effect based on deviating stimulus features.^40^ In contrast, the few studies investigating the perception of auditory features within the framework of serial dependence have reported an attractive effect toward the previously judged stimulus. Importantly, in these studies, the previous stimulus typically required a response and deviated more gradually from the current stimulus.^41^^,^^42^ Until now, both research fields studying temporal context effects in the auditory domain focused on the influence of previous sensory features but neglected the impact of the previous choice (if it was present). We aimed to test whether these contradicting perceptual context effects could be reconciled by showing repulsive auditory effects in setups of serial dependence after controlling for an attractive choice effect. The impact of choice history is also corroborated by a recent study finding an attractive serial choice effect in audition.^43^ It raises the importance of controlling for the previous choice in gaining knowledge about auditory processing. Therefore, we hypothesized finding a repulsive serial dependence of auditory features after controlling for the attractive choice effect. Evidence for two opposing processes of sensory and choice history in our auditory study would extend the well-studied effects in the visual domain and contribute to our understanding of generalized, domain-general mechanisms underlying serial dependencies.

In the present series of experiments, we examined the serial dependence of the current auditory stimulus on the preceding stimulus, i.e., speech and voice features, while considering the previous response. Participants listened to words spoken by different speakers. To induce perceptual difficulty and increase the potential of detecting opposing serial effects of stimulus and choice history, we used morphing to create a continuum between two speech sounds, specifically the vowels ‘u’ and ‘o’ contained in the word stimuli. Unlike a clear vowel, a morphed vowel is not consistently perceived as the most probable category based on its physical sensory features. This means that an ambiguous stimulus, such as 75% ‘o’, would be perceived as ‘o’ most of the time and occasionally as ‘u’ thereby leading to response variability for the identical stimulus features. If a stimulus was always perceived as the same category, stimulus features and response would be fully conflated and thus also the stimulus and the choice history. Testing the perception of intermediate steps of the morph continuum enabled us to disentangle the hypothesized opposing effects of the previous choice and stimulus features. A repulsive serial dependence effect of speech should repel the percept of the current stimulus away from the previously heard morph level, but not from the previous choice. Additionally, to test for serial dependence of voice features, we manipulated two salient and prominent voice qualities that account for considerable variability between speakers: the pitch of a voice and the vocal resonance frequencies (formants), which contribute to the distinct timbre and brightness of a voice.^44^ These two voice features influence speech classification in opposing directions. For example, on the vowel continuum between ‘u’ and ‘o’, a high pitch shifts the perception towards ‘u’, whereas high formants shift the perception towards ‘o’.^45^^,^^46^ A repulsive serial dependence of previous voice features should invert this relationship so that a high previous pitch should shift the perception towards ‘o’, while high previous formants should shift the perception towards ‘u’.

**Figure 1:**
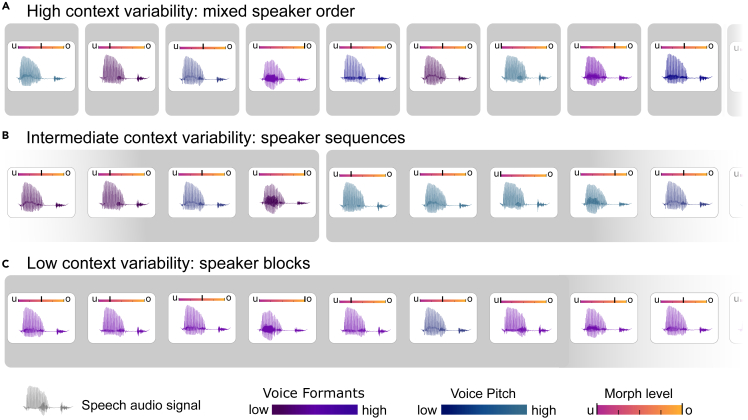
Three experimental setups (A–C) Across different experiments, we manipulated the variability of auditory voice features (i.e., pitch and formants) from (A) high context variability (mixed), (B) intermediate context variability (short sequences), to (C) low context variability, i.e., stable (block). Two voice features, the formants of a voice (purple color scale) and the pitch (blue color scale) were shifted up or down to evoke the effect of different speakers. Within each speaker, the auditory speech signal (one instance of nine word-pairs) could vary in ambiguous steps between ‘u’ and ‘o’ (morph level, orange-red color scale). Participants were asked to indicate whether they perceived ‘o’ or ‘u’ as a binary response after the audio signal via keypress.

To investigate whether the context effect found in the visual domain^28^ translates to the auditory domain, we tested whether the serial dependence on previous voice features scales negatively with increasing stability in the context environment. To that end, we manipulated the statistical context variability in which the voice properties were presented. In the most variable context, different speakers were mixed randomly (Experiment 1), in the context with intermediate variability, speakers were presented in short sequences (Experiments 2a and 2b), and in the context with the least variability, speakers were grouped in blocks (Experiment 3, see Figure 1).

## Results

### Repulsive serial dependence of stimulus features and attractive serial dependence of choice

**Figure 2:**
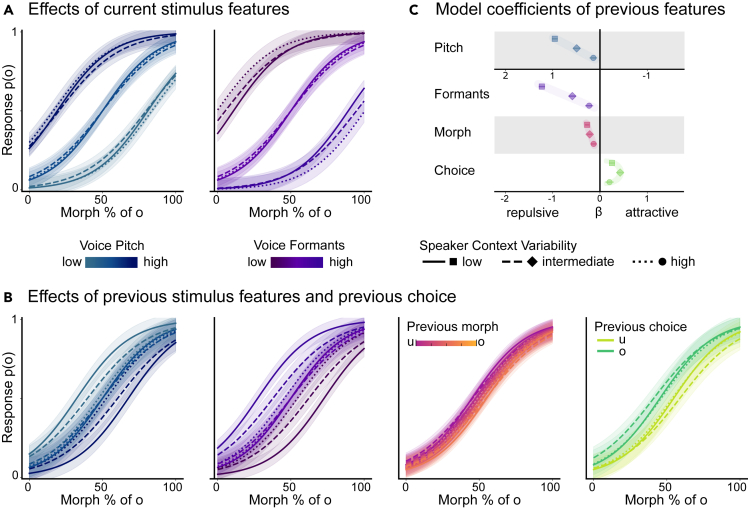
Effects of the voice, speech, and choice within different speaker variability contexts (A) Effects of current stimulus features. Influence of the current stimulus’ voice features on the speech perception of ‘o’ and ‘u’ for contexts with different levels of speaker variability (solid, dashed, dotted lines). High pitch leads to a lower probability of ‘o’ responses (first panel), whereas high formants lead to a higher probability of ‘o’ responses (second panel). (B) Effect of previous stimulus features and previous choice. Influence of the previous stimulus features and the previous choice on the perception of ‘o’ and ‘u’ in contexts with different levels of speaker variability (solid, dashed, dotted lines). The effects of the previous pitch, formants, and morph level on the perception of the current stimulus reverse: A high pitch of the previous stimulus leads to a lower probability of ‘o’ responses (first panel in B), whereas high formants of the previous stimulus lead to a lower probability of ‘o’ responses (second panel) and when the previous morph level was shifted toward ‘o’ the probability of ‘u’ responses on the current trial is higher (third panel). The previous choice exhibited an attractive effect on the perception of the current stimulus, i.e., a higher probability of responding ‘o’, when the previous choice was ‘o’ (fourth panel). All psychometric curves are plotted based on the model coefficients and their standard errors. (C) Model coefficients of previous features. The model coefficients show repulsive effects for the previous sensory features and an attractive effect of the previous choice. Repulsive effects for the sensory features of the previous stimulus (i.e., pitch, formants, and morph level) scale linearly with the levels of variability in speaker context (displayed as square, diamond, dot). The attractive effect for the previous choice exhibits a quadratic trend. All plots are based on the model coefficients and error bars (C) and shaded areas (A, B) indicate their standard errors.

To investigate the serial effects in the auditory domain, specifically of voice and speech properties, as well as the simultaneous influence of the previous choice, we recorded participants’ categorical responses, which could be either ‘u’ or ‘o’, based on morphed word stimuli spoken by different speakers. As we were specifically interested in serial effects, i.e., the influence of the preceding stimulus and choice, we accounted for the properties of the current stimulus (i.e., such as morph level, pitch, and formant manipulation) by adding them as baseline predictors to a generalized linear mixed model (GLMM). The current morph level should have a positive effect, i.e., a positive coefficient, on the perception of ‘o’, similar to the effect expected by the current formants. The baseline coefficient of the current pitch was expected to be negative since high pitch values decrease the probability of perceiving ‘o’. Next, to assess the serial dependence of previous stimulus features, i.e., the previous morph level, pitch, and formants, were added as predictors of the current choice. If a repulsive effect was apparent, the baseline effects should reverse, leading to a negative coefficient for the previous morph level and the previous formants and a positive coefficient for the previous pitch. Additionally, the previous choice was added to the model to address its possible attractive influence, shown by a positive model coefficient, on the current choice. Finally, to test for the effect of context variability, which was manipulated across three separate experimental setups, we included interactions of the context (i.e., experimental setup) with all predictors. All baseline predictors of the current stimulus showed the expected pattern of a positive slope for the current morph level (all *p* < 0.001) and the current formants (all *p* < 0.001) and a negative slope for the current pitch (all *p* < 0.001). As hypothesized, we found a reversion of these effects, i.e., a repulsive serial dependence for all stimulus-related parameters, i.e., previous morph level (low context variability −0.27 ± 0.05 [-0.36 -0.18], z = −5.02; intermediate context variability: −0.22 ± 0.03 [-0.27 -0.17], z = −8.37; high context variability: −0.13 ± 0.05 [-0.22 -0.04], z = −2.93), previous formants (low context variability: −1.23 ± 0.07 [-1.36 -1.10], z = −18.63; intermediate context variability: −0.58 ± 0.03 [-0.65 -0.52], z = −18.64; high context variability: −0.23 ± 0.05 [-0.33 -0.13], z = −4.32), and previous pitch (low context variability: b ± SE = 0.96 ± 0.06, CI [0.83 1.08], z = 15.44; intermediate context variability: 0.49 ± 0.03 [0.44 0.55], z = 16.89; high context variability: 0.14 ± 0.05 [0.04 0.24], z = 2.84; all *p* < 0.01). Note, that the positive slope for the previous pitch still indicates a repulsive effect since the baseline influence of the current pitch on the perception of the vowel ‘o’ is negative. In contrast to the stimulus features and in line with our hypothesis, we observed an attractive serial dependence of choice, attracting the current choice toward the previous one (low context variability: 0.26 ± 0.06 [0.13 0.38], z = 4.08; intermediate context variability: 0.43 ± 0.03 [0.36 0.49], z = 13.29; high context variability: 0.20 ± 0.06 [0.08 0.33], z = 73.18, all *p* < 0.01). This pattern of negative and positive effects emerged independently of the speaker variability context (see also Figure 2B). Consistently throughout all experiments, the effect of current voice features reversed for the previous voice features, i.e., a repulsive serial dependence. Also, in the speech dimension, the previous morph level influenced the current choice in a repulsive way, such that if the previous morph was an ‘o’, the current morph was more likely to be perceived as an ‘u’. At the same time, the previous choice positively shaped the current choice, attracting it toward what had previously been answered.

### Context variability influences the contrastive stimulus effect linearly and the attractive choice effect quadratically

We manipulated the speaker context variability at three different levels: speakers were presented in mixed blocks (high context variability), short sequences (intermediate context variability), or larger blocks (low context variability). To investigate the different serial effects of both sensory stimulus features and choice depending on context variability, context variability was added as a factor in the GLMM, and the predictor variables of the current and previous trials were allowed to vary freely across contexts. A linear contrast on the parameter estimates revealed that the repulsive serial dependence of voice features increased with context stability. This linear trend was apparent in both voice features of a speaker, i.e., pitch (b ± SE = −0.82 ± 0.08, z = −10.32, *p* < 0.001) and formants (b ± SE = 1.00 ± 0.08, z = −11.85, *p* < 0.001). Thus, serial dependence of the voice features scaled linearly with the manipulated context variability. Equivalently, the effect of the previous speech feature, i.e., the previous morph level, also followed a linear trend and had the greatest repulsive influence when speakers were presented in large blocks (b ± SE = 0.14 ± 0.06, z = 2.12, *p* = 0.03). Hence, all features of the previous stimulus exhibited the greatest impact in the low variability context and the weakest impact in the high variability context.

As reported earlier, the effect of the previous choice was positive throughout the different levels of context variability, but unlike the stimulus features, its magnitude revealed a quadratic effect across contexts (b ± SE = 0.40 ± 0.11, z = 3.62, *p* < 0.001). While the previous choice had the greatest impact when context variability was intermediate (speakers presented in short sequences), its effect was reduced in the low and high context variability conditions (mixed and blocked speaker presentation).

To understand this quadratic effect of context variability, we explored the possibility that participants perceived the short sequences in the intermediate speaker variability context as distinct units, similar to a chunk. In this context, participants could detect the beginning and the end of a sequence which may facilitate a chunking of physically similar trials within the sequence, in contrast to the low and high variability contexts lacking clear sequence boundaries, as the “sequences” are either very long (*n* = 207) or very short (the same speaker never appeared after itself, i.e., *n* = 1). Therefore, this may lead to a higher choice bias for within-sequence trials which are perceived as instances of the same chunk opposed to between-sequence trials that are perceived as instances of different chunks. If the increased attractive serial dependence in the context of intermediate speaker variability indeed reflects a chunking mechanism, choices within a sequence should be more influenced by the previous choices than choices made between different sequences. To test this possible explanation, we performed an additional analysis on the data of the intermediate variability context by adding an interaction term for the previous choice and the binary sequence change (i.e., whether the previous choice was made within or between sequences). Note that in the low variability context with large speaker blocks, too few between-sequence trials were available to test this hypothesis there as well. Our model revealed that indeed a significant interaction between the influence of the previous choice and sequence change was present. When the choice was made within a sequence, the attractive serial dependence became stronger (0.47 ± 0.03 [0.41 0.54], z = 13.76, all *p* < 0.001), while it was comparable in magnitude with the low and high speaker variability contexts for choices between sequences (0.25 ± 0.05 [0.16 0.34], z = 5.49, all *p* < 0.001).

### The negative effect of the previous morph turns positive when the choice is unaccounted for

**Figure 3:**
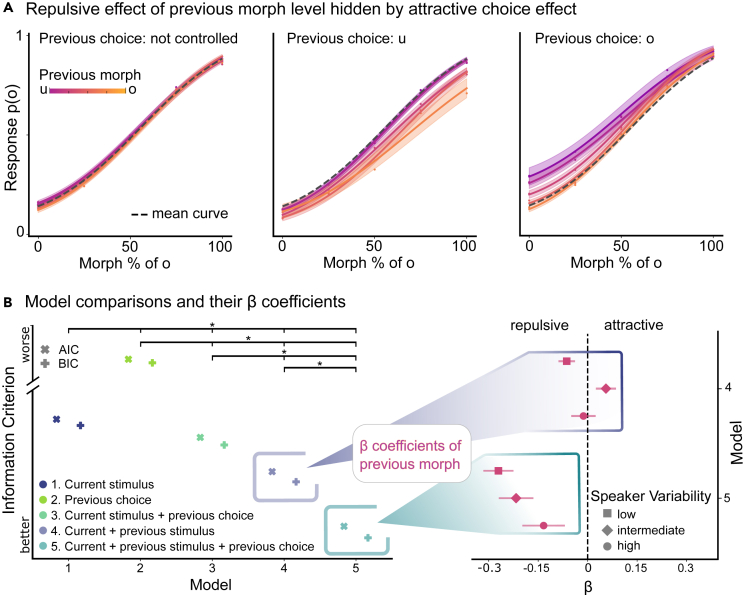
The repulsive effect of the previous morph turns more attractive when the choice is unaccounted for (A) Repulsive effect of previous morph level hidden by attractive choice effect. Without controlling for the previous choice, no repulsive effect is evident in the raw example data from the low speaker variability context (first panel). When the data are split by the previous choice, a clear repulsive effect of the previous morph level can be seen in the raw data (second and third panel). Additionally, the attractive effect of the previous choice is apparent as the curve is shifted above toward ‘u’ (i.e., most data points fall below the mean curve of the undivided data) when the previous choice was ‘u’ (second panel) and shifted toward ‘o’ (i.e., most data points fall above the mean curve of the undivided data) when the previous choice was ‘o’ (third panel). Logistic regression lines approximate the raw data points (dots) averaged across participants and their SEMs as shaded area. (B) Model comparison and their β coefficients representing the influence of the previous morph level. First panel: The current stimulus model (1.), neglecting any prior history, outperforms the pure choice history model (2.) but is surpassed by the models incorporating either the previous choice (3.) or the previous stimulus features (4.). The best-fitting model is the full model (5.) accounting concomitantly for both the previous stimulus features and the previous choice. Second panel: In the model not considering the previous choice (4.), the β coefficients for the previous morph level show a shift toward the positive (attractive) direction, indicating a conflation of the previous morph level and previous choice (top). Conversely, a distinct repulsive effect for the β coefficients of the previous morph level revealed in the model (5.), which accounts for the previous choice scaling with the speaker context variability (bottom). Error bars represent the standard error of the model coefficients. ∗ indicates ΔBIC/AIC >10.

Numerous experiments reported an attractive serial dependence of the previous stimulus features when previous decisional processes were not taken into account. This phenomenon can be explained by the intricate interplay of mutually opposing repulsive and attractive effects. When we plotted our raw data against the morph level of the previous stimulus without considering the previous choice, the repulsive effect of the previous morph level, as identified by the GLMM, was indeed not visible (Figure 3A, first panel). However, when we also accounted for the counteracting attractive serial dependence of choice, the formerly obscured negative effect of the previous morph level was revealed in the raw data (Figure 3A, second and third panel). As both effects pull the percept of the current stimulus in opposing directions with comparable weight, the positive choice effect overshadows the negative effect of the previous morph level completely.

We fitted an additional model, which deliberately excluded the previous choice as a predictor, to see how this affected the parameter estimates of the previous morph level. We expected to see a positive shift of the parameter estimates, resembing the here unaccounted effect of the previous choice on the current percept. When the previous choice was not included as a predictor in the model, the evidence for the repulsive effect of the previous morph level was mixed (Figure 3B, second panel). It varied across the contexts variabilities, so that its coefficient was significantly positive when speakers were presented in short sequences (b ± SE = 0.06 ± 0.02 [0.02 0.09], z = 3.23, *p* = 0.001), and negative when speakers were presented in blocks (b ± SE = −0.06 ± 0.03 [-0.11 -0.01], z = −2.52, *p* = 0.01) or mixed (b ± SE = −0.01 ± 0.03 [-0.07 0.04], z = −0.47, *p* = 0.64). Crucially, all parameter estimates for the previous morph level were shifted toward a more positive value and a similar quadratic trend as for the influence of the previous choice parameter can be seen.

To confirm that the model accounting for both, the influence of the previous stimulus and previous choice, was the best fit for the data, we compared five models: a model that included only the current stimulus features (Equation 1), a model that only relied on the previous choice (Equation 2), a model with the current stimulus features and the previous choice (Equation 3), a model with the current and the previous stimulus features (Equation 4), and the full model with serial dependence for both the stimulus and the choice (Equation 5). To determine the most suitable model, we used the Bayesian Information Criterion (BIC) and Akaike Information Criterion (AIC) for model comparisons, which balance the trade-off between goodness of fit and complexity. A BIC/AIC difference of Δ > 10 indicates very strong evidence in favor of the smaller value.^47^ The model that predicted participants’ responses only based on the previous choice performed the worst, and the model containing only predictors about the current stimulus improved the model fit tremendously (ΔBIC > 49000; ΔAIC > 49000). In addition to the current stimulus features, the previous choice explained further variance in predicting participant’s responses (ΔBIC > 390; ΔAIC > 420). The goodness of model fit improved further compared to the current stimulus model when including the previous stimulus features as predictors (Δ BIC > 1100, Δ AIC = 1200), and even more when adding the previous choice (ΔBIC > 2300, ΔAIC > 1200). Thus, the full model comprising previous stimulus features and the previous choice captured our data best and outperformed all other models (Figure 3B, first panel). In sum, this provides very strong evidence for serial dependence effects and that both the attractive serial dependence of choice and the repulsive serial dependence of morph level coexist and pull the percept into opposing directions.

## Discussion

In this study, we investigated the role of serial dependence in the auditory domain by manipulating the voice and speech properties of the sensory input presented in sequences with varying levels of variability. We identified two concurrent opposing serial effects: a repulsive serial dependence of the stimulus history and an attractive serial dependence of the choice history.

This finding is in contrast to many previous experiments in the visual domain, which reported an attractive serial dependence of stimulus history.^6^^,^^8^^,^^10^^,^^19^^,^^20^^,^^24^^,^^25^^,^^27^^,^^42^^,^^48^^,^^49^ However, these studies often neglected possible choice effects. We disentangled the serial dependence of perceptual features from those stemming from decision processes by applying a model that included both stimulus and choice history. In line with previous work,^30^ this comprehensive model demonstrated superior explanatory power to all other tested models, thus corroborating the plausibility of a unified account for both perceptual and decisional dependencies (Figure 3B, first panel). The direction of the serial dependence effects is in agreement with evidence from visual studies that considered the possibility of two serial effects of sensory history and choice history.^7^^,^^13^^,^^14^^,^^28^^,^^30^^,^^31^^,^^33^^,^^50^ One potential reason why many previous studies did not find a repulsive, but attractive serial dependence of sensory history may be that two concurrent history effects are working in opposing directions.^35^ This conclusion was further supported by a re-analysis of four previously published datasets which initially pointed toward an attractive effect of stimulus history, however, when the model additionally controlled for a possible effect of the previous choice, no reliable evidence for this effect was apparent.^35^ Likewise, our results demonstrate a conflated pattern in the influence of the previous morph level within the model that neglects the previous choice, resembling a combination of the repulsive sensory effect and attractive choice effect (Figure 3B, second panel). The combination of the two opposing serial effects has been determined to be additive,^30^ providing a potential explanation for why repulsive serial dependence appears to emerge from strong, salient, high-contrast, and prolonged stimuli, as the repulsive effect may exert a net dominance over the serial choice effect.^1^ Efforts have been undertaken to mitigate serial effects of choice by instructing participants to withhold their responses in some trials. This still led to attractive serial dependence in some studies^10^^,^^22^^,^^31^^,^^51^^,^^52^^,^^53^ while in others the attractive effect disappeared.^31^^,^^54^^,^^55^^,^^56^^,^^57^ The absence of an executed motor response may not necessarily rule out a (potentially implicit) perceptual decision, or the reliance on the most recent overtly realized decision, which could still be sufficient to induce an attractive serial dependence.^31^^,^^35^ This is supported by the finding that the attractive serial effect disappeared when participants were explicitly instructed to ignore the stimulus that did not require a response.^31^ Crucially, when the previous choice is accurate (i.e., the participant chose the category that matched the physical properties of the stimulus), the previous stimulus features and the previous choice are inherently and inevitably conflated. Such a conflated pattern may also be the reason that an attractive effect was found when the previous choice was correct, but when the previous choice was incorrect it turned repulsive.^58^ In sum, it is important to consider both sensory and choice history simultaneously in the analyses of serial dependence.

### The effect of context variability on repulsive sensory serial dependence

Our results indicate that the repulsive serial dependence of stimulus features, i.e., voice and speech, increases with context stability. Notably, the slope of the repulsive serial dependence of the voice features was quite steep, indicating the previous stimulus had a significantly greater influence when speakers were presented in blocks, compared to short sequences, compared to the mixed presentation (Figure 2B). This serial effect induced by sensory feature history is akin to many adaptation-like repulsive effects within and beyond the auditory domain.^17^^,^^18^^,^^59^^,^^60^^,^^61^^,^^62^ When a stimulus or a feature of a stimulus is presented repeatedly, the sensory system adapts to the statistical context with reduced responsiveness to the stimulus feature, so that the sensitivity to changes around the stimulus feature is maximised.^62^ From an information-theoretic point of view, the neural response to a stimulus is scaled by its informational value: the same stimulus carries increasingly less new information about the environment with each repeated exposure, while the informational value of a deviating stimulus scales with its amount of surprise.^63^ This is thought to be an efficient encoding strategy by the sensory system to filter relevant signals and facilitate novelty detection.^62^ In our experiment, the statistical context varied in terms of speaker variability, and the repulsive effect of the stimulus history increased with low variability likely representing a heightened informational value of the deviating information. Also in visual serial dependence research, the repulsive serial dependence of stimulus history was enhanced by decreasing context variability and further with extended sequence length.^28^ Moreover, we find that the repulsive serial effect of the speech features became slightly stronger with the context stability of voice features, although the speech variability remained constant across experimental setups. This suggests that small differences in speech features along the morphed continuum between two vowels are discriminated best in a stable voice environment. Again, this can serve as further, though indirect, evidence for an efficient encoding mechanism that facilitates more fine-grained spectral contrast detection between confusable ambiguous morph levels with repeatedly presented speakers. In line with this, increased intelligibility and faster reaction times have been demonstrated when speakers are repeated.^64^ Future research manipulating the variability of the speech context while keeping the voice context stable could corroborate whether the repulsive effect of the morph level scales steeply with speech context variability.

### The effect of context variability on attractive choice serial dependence

For the attractive serial choice effect, we observed a quadratic effect across the different levels of context variability. Note that we kept the decisional environment uncorrelated over all contexts in our experiment, as there was no predictability of the morph level, i.e., participants could not anticipate whether the next stimulus was an ‘u’ or an ‘o’. The current choice was biased strongest toward the previous choice in the intermediate speaker variability context, in which speakers were presented in short sequences. While the smaller choice effect in the high speaker variability context can be explained by the lowered subjective relevance attributed to the past choice due to lacking correlations in the statistical environment,^65^^,^^66^ this does not explain why the choice effect was also reduced in the context with low speaker variability. As an alternative explanation, the effect could be due to a chunking process during decision-making, which refers to the conscious or unconscious grouping of a set of events or stimuli to enable a more efficient encoding in comparison to when they were represented as independent elements.^67^^,^^68^ Our additional analyses support the hypothesis that in the intermediate speaker variability context, participants perceived the short sequences as discrete chunks, as we found a stronger bias toward the previous choice within a sequence. In the low variability context, speaker changes occurred very rarely, so participants most likely did not group the individual stimuli within blocks and if so, may have instead applied a metacognitive rule to not respond repetitively. When accounting for within-sequence biases, the attractive choice effect was comparable over the different levels of speaker context variability across the experimental setups. Previous research findings provided mixed results regarding the relationship between attractive choice history effects and variability of the context. While some research suggests that dependence on past responses increased with the stability of the environment,^65^^,^^66^ others indicate that the attractive choice effect diminished with greater stability.^28^ In our experiment, we found that the attractive effect of choice history remained consistent across different levels of speaker context variability, after deducting the within-sequence influence. This can be attributed to the indirect influence of voice context variability on the choice, although the decisional environment itself (speech morphs) were uncorrelated. Further research is needed to understand the factors that contribute to the modulation of attractive choice history effects, such as meta-cognition or task demands, in different environmental contexts.

### Implementation of serial dependence

The unresolved debate regarding whether “serial dependence” operates at a lower-level sensory or a higher-level post-perceptual level may partly arise from the interplay of these two opposing serial effects.^1^ Several findings indicate a higher-level origin of the attractive serial dependence, supporting our finding of an attractive decisional process. Studies using traditional analysis techniques, which disregard the previous choice, demonstrated that despite presenting stimuli with the same features, the attractive serial dependence of the stimulus features disappeared when the choice focused on a different feature.^33^^,^^54^ The finding that the attractive serial effect relies on the decision-making process supports its interpretation as a higher-level post-perceptual effect, as well as its persistence even when stimuli are merely imagined.^69^ Moreover, attractive serial dependence can only be found once the stimulus is consciously perceived and its strength scales with attention.^10^^,^^48^ Additionally, attractive serial dependence was enhanced for higher levels of confidence in a decision as well as for longer memory delays, both processes which are commonly associated with late higher-level processing.^14^^,^^29^^,^^33^^,^^70^ Furthermore, generative models implementing leaky integration of the previous choice history outperformed purely stimulus-based models^43^ underlining the great importance of previous decisions. Also, in our data, adding the previous choice as another predictor to the model explained the data significantly better. In most experiments, including ours, only the sensory input and the resulting response output are observable, leaving the intermediate processing stages undisclosed. Since the behavioral response is the overt manifestation of perception, it remains unclear whether the attractive effect already affects processing at the perceptual level in a top-down manner, rather than emerging during post-perceptual processing. The neural signatures of serial dependence may reveal further insights into its implementation. The two opposing serial processes appear to map on different brain networks: areas in early sensory regions were shown to be involved in repelling perception away from sensory history,^32^^,^^71^^,^^72^ whereas frontoparietal regions were involved in the attractive effect of the previous choice.^72^^,^^73^ Correspondingly, several studies demonstrated this memory trace of the previous trial at different timepoints relative to the current stimulus presentation in human EEG signals.^7^^,^^12^^,^^74^^,^^75^^,^^76^ The accuracy of decoding the neural signature of the past response correlated positively with the strength of observed serial dependence reflecting the behavioral relevance of this neural representation of past experience.^74^^,^^76^ As most of the mentioned studies focused on the possibility to decode previous information from the current trial, the exact neural mechanism of how previous and current information are combined, is still unclear. Some insight comes from spiking activity analyses from neurons in the mouse brain where a strengthened correlation between the neural pattern and behavioral choice increased across the ascending cortico-thalamic hierarchy.^77^ This indicates a possible integration of the encoded repulsive sensory signal with attractive top-down mechanisms that finally results in an (attractive) choice behavior.

### Why is there an attractive serial dependence?

Intriguingly, the serial effect consistently manifested as attractive across different experiments - regardless of whether it was interpreted as the net dominance of an attractive decisional effect over a repulsive sensory effect^7^^,^^13^^,^^14^^,^^28^^,^^31^^,^^32^^,^^35^^,^^78^ or as an attractive sensory-perceptual effect.^6^^,^^10^^,^^22^^,^^24^^,^^25^^,^^26^^,^^27^^,^^49^ An overall attractive effect has been shown to be difficult to eliminate, as it persists even in entirely uncorrelated experimental contexts, despite impairing performance.^65^ This raises the question why there is attractive serial dependence. A possible explanation is that in everyday perception, the past is typically correlated with the future which could lead to default top-down prediction that the current precept will resemble the previous one. Once this statistical regularity is learned,^79^ small negligible variabilities in the sensory input may be smoothed out thereby achieving an adaptive stable representation of the environment.^3^ Even though a recent study found diminished attractive serial dependence of choice history when introducing correlations in the decisional context, the analysis of the error responses showed that participants made more correct choices.^28^ This suggests when deliberately introducing a positive bias toward the previous choices in an experimental context, comparable to the real-world environment, attractive serial dependence renders perception more precise and veridical. In sum, serial dependence may involve a balanced interplay between a sensory encoding mechanism that is sensitive to detecting changes which results in repulsive sensory serial dependence, and the attractive decoding mechanism of this repulsive sensory evidence to filter out less meaningful variations leading to a stable attractive serial dependence of choice.

### Comparing serial dependence in the visual and auditory modality

In the current study, we examined the effect of the stimulus and choice history on the perception of auditory stimuli. Pre- and post-perceptual mechanisms in the auditory domain may be intrinsically different from the visual domain, as temporal statistics of events in the external world differ for both modalities. The auditory signal is a wave that conveys information by variability over time and thus inherently needs temporal change, whereas changes in the visual environment are typically sparse and slow,^80^ i.e., there is more variability in the auditory domain and especially in spoken word recognition these fluctuations are meaningful, whereas in the visual domain sensory input may change on a comparative slower timescale which renders small changes as uninformative. Our results complement the traditional serial dependence literature demonstrating attractive effects of the stimulus history,^1^^,^^8^^,^^10^ by reconciling a repulsive effect of the previous stimulus features and an attractive effect of the previous choice (as previous studies^7^^,^^13^^,^^14^^,^^28^^,^^35^).

Nevertheless, the pattern of results we observed in our study, i.e., opposing effects of sensory and choice history, could also be explained by inherent differences in the auditory and visual modality. For auditory stimuli the weighting of top-down influence and bottom-up sensory evidence may be shifted, so that in contrast to visual stimuli, small deviations in auditory input are weighted stronger, leading to a higher likelihood of observing a repulsive effect. A recent comparison of visual and auditory serial dependence in duration perception showed no difference in either magnitude of the repulsive sensory or the attractive choice effect between the two sensory domains,^50^ suggesting that the sensory processing for vision and audition likely relies on similar mechanisms. Although the temporal statistics in the external world of both modalities are inherently different, the pattern of serial dependence is similar, showing repulsive sensory processing together with an attractive decisional effect. Hence, our findings hint at a shared mechanism operating in both vision and audition. Similar to our study, opposing serial effects in agency^81^ and tactile vibration perception^82^ support the speculative notion of a domain-general mechanism underlying serial dependence. Accounting for both sensory and choice history across several modalities will elucidate whether a general, modality-invariant mechanism for serial dependence exists.

### Limitations of the study

The two-alternative forced choice task implemented here used stimuli that varied in voice (pitch and formants) and in speech features (u-o-continuum) while the binary choice discriminating between ‘u’ and ‘o’ was collected as a response measure. This categorical task may not fully encompass the response variability that may span the entire continuous morph spectrum,^83^ since it has been suggested that speech processing may involve a gradient mapping^84^ rather than a strict categorical mapping. However, a recent study design with a non-categorical pitch reproduction task revealed the same repulsive effect of the previous stimulus with an attractive effect of the previous choice.^7^ Hence, while the magnitude of our effects could be under- or overestimated by the dichotomous response scale, the direction of our observed effects, as well as the relation between the speaker variability contexts should be unaffected by our task. Future studies may test graded, continuous response options similar to the rating responses in the visual domain,^10^ presumably leading to more differentiated error patterns.

In our study, we manipulated voice features related to speech perception simultaneously with speech features. However, we measured speech perception which captured the impact of voice features only indirectly. This was possible because we previously established the direction of voice influence, i.e., the pitch and the formant frequencies, on the perception of vowel contrast.^46^ As hypothesized, the repulsive effect of those voice features was shown by reversed perceptual effects for current and previous voice features. Given that similar repulsive effects were noted in other studies where the response dimension directly involved pitch, it is plausible that our results generalise.^7^ Further, the experimental design choice to focus only on one dimension (the speech dimension) was necessary since serial dependence is thought to be feature-specific and disturbed by decisions associated with a different feature dimension.^7^^,^^33^

The voice features in our experiments only varied in three steps from lower to mid-range, to higher than average. Though improbable, it is still possible that smaller, more nuanced differences in the voice features would have revealed an effect in the opposing direction, since it has been argued that attractive serial dependence operates over a limited range of feature (dis-)similarity.^1^ In our study, a gradual manipulation of the voice features would have interfered with the speaker variability context as the speakers would not have been discriminable anymore. A more fine-grained manipulation of the voice features could be addressed by future studies focusing on the serial effect of voice features in the absence of context variability.

One further consideration in our study is that in the intermediated speaker variability condition, grouping within the sequence may have been strengthened by the presentation of static face images. This could have contributed to a stronger grouping within than between a sequence, even though the visual information was uninformative of the speech information, and therefore should not affect the choice.

Our design intentionally used a stable mapping of keys and responses because trial-wise key switches would have been confusing for participants, especially in an online experimental environment. Thus, the attractive effect of the previous choices was paralleled by the history of motor activity since in our experiment the choice and motor response are inseparable. Nevertheless, motor history should only have dampened the attractive choice effects because previous studies have shown that motor response history either had no influence on the attractive effect of the previous choice^66^ or that it had a repulsive effect.^7^^,^^85^

In conclusion, our study showed opposing serial effects on auditory vowel perception with repulsive sensory and attractive decisional effects. Extending findings from visual perception research, we identified parallel serial effects in the auditory domain, with intriguing context variability effects which suggest an adaptive sensory mechanism for repulsion and a higher-level mechanism for attraction.

## STAR★Methods

### Key resources table

**Table:** 

REAGENT or RESOURCE	SOURCE	IDENTIFIER
**Deposited data**		

Experimental data	This paper	https://doi.org/10.17605/OSF.IO/9TJ57
Stimuli	This paper	https://doi.org/10.17605/OSF.IO/9TJ57

**Software and algorithms**		

R2023	R Core Team	https://www.r-project.org/
RStudio	RStudio Team	https://posit.co/products/open-source/rstudio/
lme4	Bates et al.^86^	https://doi.org/10.18637/jss.v067.i01
Python 3.10	Python Software Foundation	https://www.python.org/
MATLAB	Mathworks	https://mathworks.com/
STRAIGHT	Hideki Kawahara	https://github.com/HidekiKawahara/legacy_STRAIGHT
Praat	Paul Boersma and David Weenink	https://www.fon.hum.uva.nl/praat/
Prolific	Prolific	https://www.prolific.com/
Pavlovia	Open Science Tools	https://pavlovia.org/
jsPsych 6.3	Josh de Leeuw	https://www.jspsych.org/6.3/
Code for analyses	This paper	https://doi.org/10.17605/OSF.IO/9TJ57

### Resource availability

#### Lead contact

Further information and requests for resources should be addressed to the lead contact, Carina Ufer c.ufer@uke.de.

#### Materials availability

The dataset, code and stimuli generated in this study have been deposited online (see key resources table). No other materials were produced by this study.

#### Data and code availability

• Experimental data and the stimuli have been deposited at OSF and are publicly available as of the date of publication. DOIs are listed in the key resources table.
• The original code to reproduce the results is publicly available at OSF and the DOI is listed in the key resources table. The code for the online experiment and the code for plotting the results is available from the lead contact upon request.
• Any additional information required to reanalyze the data reported in this paper is available from the lead contact upon request.

### Experimental model and study participant details

We conducted a series of four online experiments, in which we investigated the influence of stimulus and choice history on the current auditory percept. Across four experiments, we recruited 179 healthy participants (experiment 1: 30 participants (17 males, 13 females, 18-60 years, M_age_ = 28.33, SD_age_ = 9.26); experiment 2a: 33 participants (16 males, 17 females, 19-40 years, M_age_ = 29.76, SD_age_ = 5.99), and experiment 2b: 87 participants (43 males, 44 females, 19-39 years M_age_ = 27.53, SD_age_ = 5.20); experiment 3: 30 participants (19 males, 11 females, 18-57 years, M_age_ = 29.0, SD_age_ = 9.63). Participants were screened to have normal hearing, no neurological or psychiatric illnesses, and German as their first language. Data from experiments 1 and 3 were existing datasets from previous experiments.^46^ All participants were recruited via the online platform Prolific and were rewarded 7.50 £ per hour for experiments 1, 3, 2a, and inflation-adjusted 12.00 £ per hour for experiment 2b. The sample size was based on previous work.^46^ Written informed consent was given by each participant at the start of the experiment. The local ethics committee approved the study.

### Method details

#### Stimuli

Across all four experiments, we used the same audio stimuli. These were the following real word pairs in German, that phonetically only differ between the central ‘u’ or ‘o’ vowel:

(Brut / Brot (brood / bread); Bug / Bog (bow / bent); Fuhr / Vor (rode / before); Huhn / Hohn (chicken / derision); Kur / Chor (cure / choir); Ruhm / Rom (glory / Rome); Schuh / Show (shoe / show); Tut / Tot (toot / dead); Zug / Zog (train / drew).

The mean duration of the stimuli was 642 ms (SD = 67 ms). Between the u/o endpoints, morphs were created in 5 steps using the STRAIGHT vocoder in MATLAB, keeping the pitch contour constant at the value of the /u/ endpoint. All audio stimuli were recorded by a professional female speaker. To create the average voice sound based on variance in the population we adjusted the average pitch of the initial recording to 170 Hz and modified the formant frequencies of the original voice by reducing them by 7%. To create the four additional speaker identities, the average speaker's voice was shifted up or down in either pitch or formant frequencies using Praat's "change gender" routine. The shift factors were 1.146 or 1/1.146 = 0.873 for the high and low formant frequency speakers, and 1.24 or 1/1.24 = 0.806 for the high and low pitch speakers, respectively. These factors were chosen with a pitch/formant frequency ratio of approximately 1.6 which is the relative importance of these factors when determining different voice identities.^87^ The differences between each of the four created voices to the average speaker were chosen to induce reliable percepts of different speakers, i.e., less than 25% formant frequency difference and 45% pitch difference.^87^ In general, the pitch variation was reduced with a factor of 0.2, to increase the perceived similarity within test stimuli.

#### Apparatus

We conducted a series of four online experiments in which participants listened to sequences of spoken words via headphones (which was tested via a headphone test at the beginning of each experiment^88^). Overall experiments, participants indicated their responses by pressing the respective keyboard key (‘u’ / ‘o’). If participants did not respond in time, a 1500 ms long prompt informed them that they had been too slow. After each auditory stimulus, there was a minimum of 500 ms of silence up to the maximum response time.

#### Experiment 1 - High speaker variability

In experiment 1, the spoken words were presented in random order with the two constraints that the same speaker could not be followed by itself and the same word could not appear consecutively by two different speakers. For each participant, there were 828 trials, resulting from 9 words x 4 repetitions for the 4 shifted speakers with 5 morph levels per word, and for the average speaker with 3 morph levels per word. Thus the probability of occurrence for the average speaker was 13%. On the screen, participants were presented with the response options ‘u / o’ each trial for which they were given 5000 ms time to answer. The experiment was split into 8 blocks with a break of 15 s in between. On average, participants took 21.39 min (range 18.19 to 25.54 min) to complete all experimental trials, without the time allocated for reading and accepting the informed consent and undergoing auditory checks at the beginning of the experiment.

#### Experiment 2 a + b - Intermediate speaker variability

##### Experiment 2a

Participants were presented with sequences of words of varying length (7, 8, or 9 words) by the same shifted speaker, however, one word per sequence was exchanged to be spoken by the average speaker. The average speaker was presented at every position once (except the first one to establish context). Each sequence length was presented six times per speaker. This led to a total of 576 trials ((7+8+9) words x 6 repetitions x 4 speakers) per participant, of which 72 were spoken by the average speaker. Thus the probability of a word spoken by the average speaker was 12.5%. Because of the few trials, we restricted the average speaker words to be the most ambiguous 50% morph level. The order of the speakers was pseudo-randomized for each participant and constrained such that between sequences the same speaker could not be followed by itself. Within a sequence, each word could only appear once, and between sequences the same word could not be repeated. Each trial prompted a response and lasted 2500 ms because in experiment 1 participants' response times very rarely exceeded 2000 ms.

During auditory word presentation, one of four faces was shown to be associated with each speaker randomly. To further ensure participants' engagement, we additionally introduced 8 catch trials in which participants had to respond with the space key to a red dot on the screen, which in return disappeared again. If more than 3 catch trials were missed, the experiment would terminate automatically, which did not happen for any participant. On average, participants took 23.25 min (range 20.50 - 27.36 min) to complete all experimental trials.

##### Experiment 2b

Experiment 2b was equivalent to experiment 2a except that the sequence length was fixed to 7 words. This led to a total of 504 trials (7 words per sequence x 18 sequences x 4 speakers) each prompting a response. Over the course of the experiment, the average speaker was presented at each of the 7 sequence positions at least twice, and at 4 positions three times, resulting in 18 trials per speaker. On average, there was approximately a 14% chance of a word being spoken by the average speaker. The order of the speakers was pseudo-randomized over the experiment. Participants took on average 21.46 (range 18.52 - 28.42 min) to complete all experimental trials.

We merged both sequence experiments 2a and 2b because they were based on identical tasks and procedures. There were no differences in the direction of the repulsive stimulus history effects and the attractive choice history effect and also the magnitude of the effects was comparable for all parameters (all *p* > 0.15) except for the history effect of the pitch, which was stronger for experiment 2b (b ± SE = 0.14 ± 0.06, *p* = 0.02).

In the two sequence experiments, we presented images of faces during the spoken words to address a different research question of another study which is reported here for transparency only. One of four faces was shown to be associated with each speaker, assigned randomly across participants. In the middle of both experiments, participants completed a face-voice association training, which was part of the research question of another study and is reported here for transparency only. During the face-voice association training participants listened to the audiobook “Alice im Wunderland” ("Alice in Wonderland”) read by the four speakers. In the first part, they listened passively while in the second active part, they had to actively indicate which speaker they were currently listening to via a mouse click and received feedback. At the end of the experiment, a test of whether participants learned the face-voice associations was conducted. This training did not affect the serial dependence effects of the current study. We assessed if there were differences in serial dependencies from before to after the face-voice association training with an extended GLMM. There were no differences between the two parts that could not be explained better by a linear time trend over all blocks of the experiments (both BIC and AIC > 280). Even though there were small significant differences throughout the experiment attenuating the serial effect that the previous morph and the previous choice had on the current trial, the estimated effect of the previous morph at the end of the experiment was still negative (b ± SE = -0.11 ± 0.04, CI [-0.19 -0.04], z = -2.89) and the estimated effect of previous choice at end of the experiment was still positive (0.36 ± 0.04 [0.29 0.44], z = 9.11, both *p* < 0.01), the pattern of repulsive sensory history and attractive choice history remained stable.

#### Experiment 3 - Low speaker variability

In total, there were 828 trials for each participant, resulting from 9 words x 4 shifted speakers x 5 morph levels x 4 repetitions (720), and additional for the average speaker 9 words x 3 morph levels x 4 repetitions (108). The experiment was split into four blocks based on the four speakers, each comprising a total of 207 trials, of which 180 word stimuli were spoken by the shifted speaker, randomly interspersed with 27 words spoken by the average speaker. Thus the probability of a word spoken by the average speaker was 13%. Neither two words by the average speaker, nor the same word could repeat itself. Note here that with this experimental setup, the probability of a word being spoken by the average speaker was nearly identical over the three variability conditions across the three experimental setups and only the speaker context changed. In the middle and between each block there was a 15 s break. For every trial, participants were presented with the question ‘u / o’ for which they had 5000 ms time to answer. They took on average 22.21 min (range 19.95 - 25.67 min) to complete all experimental trials.

### Quantification and statistical analysis

All statistical analyses were performed using RStudio version 2023.6.0.421 with the R version 4.2.0^89^ and the lme4 package for generalised linear mixed models.^86^

Participants' responses were manually checked by counting their consecutively repeating or alternating choice patterns and by plotting psychometric functions. If a random response pattern was obvious, participants were confronted with it via a direct message and could voluntarily return their submission. In total, 7 participants withdrew their participation. We excluded one additional participant who responded 34 times before the audio stimulus ended, thus indicating low effort.

Before analyses, participants’ responses were cleaned for outliers. We considered trials in which participants responded under or over 3 SD of their mean reaction time as outliers, which led to the exclusion of a maximal 4% of trials per participant.

As the responses were binary a logistic linking function was specified. The response variable was coded as 1 if ‘o’ was answered and 0 if ‘u’ was answered. We hypothesised that the responses were influenced by the morph level, the pitch, and the formant frequencies of the voice of the current stimulus, but crucially also by the previous morph level, the pitch, and the formant frequencies of the voice of the previous stimulus, and the previous response. A similar analysis approach of using GLMMs to investigate serial dependence in the visual domain was recently applied.^13^^,^^78^ In accordance with the literature, we followed a maximal approach including as many random effects per participant as possible while retaining model convergence.^90^ We first included participant random slopes for all fixed effects and their interactions and reduced the model stepwise by prioritising the fixed effects of interest until the model successfully converged. This approach led to the following formula:

$$Response'o'∼morph\;level\times context+pitch\;of\;the\;voice\times context+formant\;frequencies\;of\;the\;voice\times context+pitch\;of\;the\;voice_{prev}\times context+formant\;frequencies\;of\;the\;voice_{prev}\times context+morph\;level_{prev}\times context+choice_{prev}\times context+\left(1+morph\;level|word\right)+\left(1+morph\;level+pitch\;of\;the\;voice_{prev}+formant\;frequencies\;of\;the\;voice_{prev}+morph\;level_{prev}+choice_{prev}|participant\right)$$

All fixed effect predictors, except the factorial context predictor, were rescaled and centred to fall within the range from -1 to 1 to ensure comparable effect sizes.^91^ The context variable was dummy-coded and the blocked speaker condition was defined as the reference level. For all post hoc tests and for the estimation of 95% confidence intervals (corresponding to a significance level of *p* = 0.05) and *p*-values the emmeans package was used.^92^ As a second step, contrasts over the GLMM parameter estimates were applied to characterise the magnitude of the effect over the different contexts. The linear and quadratic effect of the parameter estimates obtained from the GLMM was assessed using custom contrasts (linear: [-1, 0, 1] or quadratic [-0.5, 1, -0.5]) on the estimated marginal means per experiment.

For model comparison, four alternative models to the full model were tested: (1) the stimulus model which contained information about the current stimulus, (2) the previous choice model with the previous choice as the only predictor, (3) the model which contained additional to the previous choice the current stimulus features as a predictor, and (4) the previous stimulus model which included predictors about the current and the previous stimulus features. For comparability reasons, we kept the random effects structure similar across models and therefore also fitted the full serial dependence model (5) with the reduced random effects structure.

(Equation 1) Responseo′′∼morphlevel*context+pitchofthevoice*context+formantfrequenciesofthevoice*context+(1|word)+(1|participant)

(Equation 2) Responseo′′∼previouschoice*context+(1|word)+(1|participant)

(Equation 3) Responseo′′∼morphlevel*context+pitchofthevoice*context+formantfrequenciesofthevoice*context+previouschoice*context+(1|word)+(1|participant)

(Equation 4) Responseo′′∼morphlevel*context+pitchofthevoice*context+formantfrequenciesofthevoice*context+previousmorphlevel*context+pitchofpreviousvoice*context+formantfrequenciesofpreviousvoice*context+(1|word)+(1|participant)

(Equation 5) Responseo′′∼morphlevel*context+pitchofthevoice*context+formantfrequenciesofthevoice*context+previousmorphlevel*context+pitchofpreviousvoice*context+formantfrequenciesofpreviousvoice*context+previouschoice*context+(1|word)+(1|participant)

For model comparison, we chose to compare both, Akaike information criterion (AIC) and Bayesian information criterion (BIC), because both information criteria may favour different models as the BIC, different from the AIC, penalises more complex models additional to the number of predictors for the number of observations, thereby preferring simpler models over more complex ones compared to the AIC. The difference in BIC and AIC were interpreted in line with the guidelines for model selection so that a difference > 10 was judged to be very strong evidence in favour of the model with the smaller value.^47^

We conducted an additional analysis to test for differences in the dependence on the previous choice in the intermediate speaker variability context within a sequence versus between sequences. We filtered out the corresponding data and added the interaction between the previous choice and the factor variable whether the current decision was within or between sequences. Again we followed the maximal approach for model fitting, which resulted in the following model:

$$Response'o'∼morph\;level+pitch\;of\;the\;voice+formant\;frequencies\;of\;the\;voice+morph\;level_{prev}+pitch\;of\;the\;voice_{prev}+formant\;frequencies\;of\;the\;voice_{prev}+choice_{prev}\times within/between\;sequnece+\left(1+morph\;level|word\right)+\left(1+morph\;level+morph\;level_{prev}+choice_{prev}|participant\right)$$
